# Biogeographical Differentiation and Key Drivers of the Home-Field Advantage in Cross-Habitat Litter Decomposition: A Global Meta-Analysis

**DOI:** 10.3390/plants15172716

**Published:** 2026-09-04

**Authors:** Tianjiao Mei, Xingbing He, Yonghui Lin, Zaihua He, Xiangshi Kong

**Affiliations:** 1 Hunan Provincial Key Laboratory of Ecological Conservation and Sustainable Utilization of Wulingshan Resources, School of Life Sciences, Jishou University, Jishou 416000, Chinakongxiangshi@126.com (X.K.); 2 School of Life Sciences, Jishou University, Jishou 416000, China

**Keywords:** home-field advantage, litter decomposition, biogeographical differentiation, climate type, vegetation type, meta-analysis

## Abstract

Global climate change and human activities have jointly exacerbated habitat fragmentation and expanded ecotones, significantly increasing the frequency and intensity of cross-habitat litter decomposition. The phenomenon whereby litter decomposes faster in its habitat of origin (“home”) than in other habitats (“away”) is known as the home-field advantage (HFA) effect. In-depth exploration of its core mechanisms and key drivers is crucial for revealing the spatial heterogeneity of global carbon and nitrogen cycles and for refining the theory of substance cycling in forest ecosystems. This study integrated 1410 observations from 102 published studies and used meta-analysis to systematically evaluate global patterns, biogeographical differentiation, and key drivers of the HFA effect. The results show that the HFA exhibits significant biogeographical differentiation and is driven by multiple factors. Globally, we found a significant positive HFA (*LnRR* = 0.0576, *p* < 0.05), with decomposition-related metrics (e.g., mass loss, decomposition rate) being 5.9% higher at home than away. The strength of the HFA effect shows significant dependence on climate and vegetation types. Regarding vegetation type, significant home-field advantages were observed for coniferous forests (10.15%), deciduous broadleaved forests (8.31%), and evergreen broadleaved forests (6.57%), while no significant differences were detected for shrublands or grasslands. Regarding climate type, subtropical (6.46%), temperate (9.20%), and cold/highland climates (9.53%) exhibited significantly higher home-site effects, whereas tropical and arid/semi-arid climates showed no significant differences. All analyses demonstrated substantial heterogeneity (I^2^ = 65.5–98.3%). This study also systematically analyzed the associations of three core factor categories (environmental factors such as elevation and precipitation, litter chemical composition, and soil chemical properties) with HFA effect. Among these factors, Litter C/P and Litter N/P were verified as significant positive regulatory factors governing the HFA effect. In conclusion, the driving mechanism of the HFA exhibits systemic characteristics that manifest as the interactive and synergistic effects of multiple factors rather than the independent control of a single factor. Specifically, climate and vegetation types jointly dominate the formation of its macro-geographical patterns, while local abiotic factors (e.g., altitude, precipitation) and litter properties (e.g., chemical composition) further and precisely regulate the strength of the HFA effect through complex interactions. Based on a systematic evaluation using meta-analysis, this study provides an important theoretical basis and data support for understanding the driving mechanisms of the HFA effect in cross-habitat litter decomposition across different ecosystems.

## 1. Introduction

Forests are the largest organic carbon pool in terrestrial ecosystems, storing 80% of the global aboveground organic carbon and exerting a key influence on global carbon cycles [1]. Litter comprises all organic residues that naturally detach from aboveground plant organs and fall back to the ecosystem surface [2]. As the primary pathway for nutrient return, plant litter decomposition is recognized as central to forest carbon cycling and nutrient redistribution [2]. Although at broad geographic scales litter decomposition is controlled by climate and litter quality [3,4], accumulating empirical studies demonstrate equally critical regulation by small-scale biological drivers, especially the decomposer community [5,6,7]. Soil fauna significantly promotes litter decomposition by an average of 27% across global and biome scales and is widely considered to be a primary driver of litter decay and nutrient transformation [8,9]. Studies have demonstrated that plant litters derived from distinct habitats exhibits significant divergence in their nutrient composition [10]. Under conditions of nutrient competition within the soil biota, local microbial decomposer communities (bacteria and fungi) preferentially adapt to native litter substrates. This adaptation is manifested via the secretion of specific hydrolytic enzymes, which efficiently target and degrade the unique biochemical compounds of native litter. This process accelerates litter decomposition in its original habitat compared to other habitats, a phenomenon known as the home-field advantage (HFA) [11,12,13,14,15]. Milcu et al. [11] and Ayres et al. [12] demonstrated in a large-scale meta-analysis that local leaf litter decomposes on average 8% faster than non-local litter, which is considered ecologically meaningful in litter decomposition studies.

However, despite the widespread observation of HFA, its occurrence is highly variable and context-dependent [16,17,18,19]. This variability is evident in its effect sizes, which can be positive, negative, or neutral, and also in the differing magnitudes of the effect observed across various litter types [14]. Most reported cases of HFA have been observed either between highly disparate ecosystems [20,21,22] or in reciprocal transplant experiments involving litters of substantially contrasting qualities [21,23,24,25,26]. Conversely, when litter enters a non-native habitat with similar litter quality, HFA is not detected due to the absence of specialized microbial communities [17]. Based on this pattern, Veen et al. [27] predicted that the likelihood of observing HFA increases with greater differences between the “home” and “away” plant communities.

Assessing the HFA provides critical insights into the dynamics of litter decomposition in native versus foreign habitats and has been empirically tested across various ecosystems through reciprocal litter transplantation experiments [28,29]. Whether an HFA is observed appears to be attributed to variation in the main factors controlling decomposition, including climate [27], litter quality [12], and the decomposer community [21], as well as to more context-dependent processes such as litter site affinities [30], substrate–matrix interactions [19], and phenology–substrate matching [31]. Recent evidence suggests that initial litter chemical traits and external nutrient inputs may play important regulatory roles in HFA. For instance, Li et al. [32] reported that, along an N addition gradient of 0–50 g N m^−2^ yr^−1^, litter C:N and C:P ratios decreased significantly with increasing N addition rates, and the home-field advantage effect was concurrently enhanced. Similarly, Lin et al. [33] found that, at an N addition rate of 47 kg N ha^−1^ yr^−1^, the decomposition rate of *Pinus massoniana* litter (initial C:N ratio of 86.41, lignin:N ratio of 111.35) in its home environment was significantly enhanced, while no significant promotion of decomposition was observed for *Quercus variabilis* litter (initial C:N ratio of 61.73, lignin:N ratio of 40.69) with the same N addition treatment. Moreover, two meta-analyses have reported that HFA effects are influenced by litter quality indicators: Wang et al. [13] found a negative correlation between lignin:N ratio and HFA (r = −0.403), while Veen et al. [27] reported that HFA significantly decreased with increasing C:N ratio, collectively suggesting that higher-quality litters (lower C:N and lignin:N ratios) tend to exhibit stronger HFA effects. The chemical complexity and recalcitrance of the three litter types, ranked from highest to lowest, are rhododendron, pine, and grass, based on C:N ratios [24] and previously reported in situ decomposition rates for identical or very similar litter types [34,35,36]. Correspondingly, the rhododendron and pine inocula displayed stronger home-field advantages on their respective home litters, while the grass inoculum exhibited a weaker HFA on grass litter. This indicates that greater litter chemical complexity and recalcitrance are associated with a more pronounced HFA [24]. Soil fauna plays important roles in plant litter decomposition, through three primary mechanisms: (1) physical fragmentation of litter to increase surface area for microbial colonization, (2) dispersal of microbial propagules, and (3) stimulation of microbial activity via trophic interactions. Soil microorganisms contribute to HFA through substrate-specific enzymatic adaptations [11]. These processes collectively amplify HFA-driven litter mass loss [37,38]. Based on the aforementioned studies, we propose the following hypotheses: (1) the home-field advantage (HFA) effect in litter decomposition exists overall at the global scale, with the decomposition rate of native litter being generally significantly higher than that of non-native litter across various forest ecosystems; (2) the intensity of the HFA effect in litter decomposition is jointly regulated by litter quality traits and key environmental factors such as climatic conditions and soil properties, with complex interactive effects among these factors.

The ongoing influences of global climate change and human activities such as excessive deforestation and logging, large-scale burning of fossil fuels, and destruction of grassland vegetation increase the likelihood that plant litter may decompose in ecosystems with distinct microbial communities rather than in its native environment. The functional specialization of decomposer communities, shaped by long-term adaptation to specific litter types and local habitat conditions, is considered to be a key mechanism driving the HFA in litter decomposition. In this context, our study integrates existing global observational data and employs meta-analysis to address two central questions: (1) whether there is a significant home-field advantage effect in litter decomposition at the global scale and what the magnitude of this effect is; and (2) what key factors influence the home-field advantage effect and how these factors regulate this effect. The findings will provide a scientific basis for accurately predicting plant nutrient return pathways and ecosystem carbon budgets in the context of global environmental change. Furthermore, exploring the strength and direction of the HFA provides theoretical support for understanding the role of plant–soil feedback (PSF) in regulating litter decomposition in global change scenarios.

## 2. Methods

### 2.1. Data Collection and Selection

From the relevant studies retrieved, a total of 102 published papers were identified for inclusion, which included 1410 cases (Supplementary Information Table S1). We searched articles published before 12 January 2026 using the China National Knowledge Infrastructure (CNKI, https://www.cnki.net/, accessed on 12 January 2026), Web of Science (https://www.webofscience.com/, accessed on 12 January 2026), Google Scholar (https://scholar.nq69.top/scholar.html, accessed on 12 January 2026), Elsevier (https://service.elsevier.com/, accessed on 12 January 2026), Wiley (https://onlinelibrary.wiley.com/library-info/products/journals, accessed on 12 January 2026), SpringerLink (https://link.springer.com/, accessed on 12 January 2026), Science (https://www.science.org/, accessed on 12 January 2026), Nature (https://www.nature.com/, accessed on 12 January 2026), BaiLian (https://www.blyun.com/, accessed on 12 January 2026), and other Chinese or English databases. The following search strategy was applied: (“home-field advantage” OR “home field advantage” OR “HFA” OR “home effect”) AND (“litter decomposition” OR “litter decay” OR “mass loss” OR “decomposition rate”) AND (“carbon” OR “soil respiration” OR “carbon cycling” OR “carbon release” OR “carbon flux”). For the Chinese databases (CNKI and BaiLian), equivalent Chinese search terms were used. Additional keywords including organic matter degradation, organic carbon decomposition, carbon efflux, and carbon emission were also used as supplementary search terms. This meta-analysis strictly followed the Preferred Reporting Items for Systematic Reviews and Meta-Analyses (PRISMA) guidelines to ensure the transparency and reproducibility of the data collection process [39]. All of the articles were selected based on the following criteria: (1) the litter mass loss and decomposition rate for both home and away environments in the reciprocal transplant experiment should be reported; (2) review articles, meta-analyses, and other non-original research articles were excluded; and (3) the means, standard deviations, and sample sizes were reported or could be determined.

**Figure 1:**
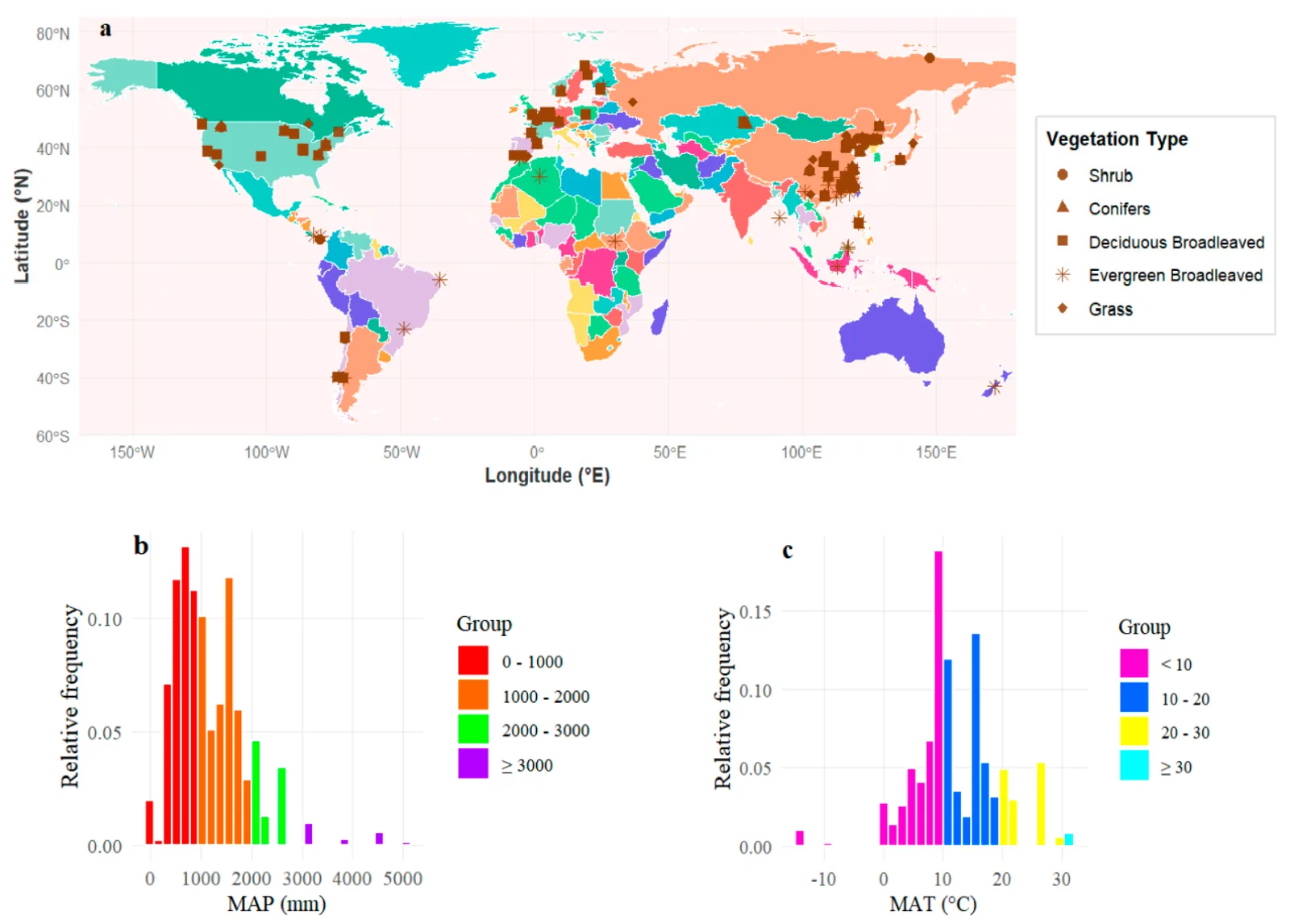
Coordinates of study sites and histograms. (**a**) Geographical location of the 1410 study cases included in this meta-analysis; (**b**) histogram of annual mean precipitation (MAP); (**c**) histogram of annual mean temperature (MAT).

Then, we extracted the means, standard deviations, and sample sizes for each target variable from the tables, figures, main text, or Supplementary Materials of the selected articles. The data presented in figures were digitized using WebPlotDigitizer [40]. We also recorded the longitude, latitude, altitude, mean annual precipitation (MAP, mm yr^−1^), altitude (m), mean annual temperature (MAT, °C), vegetation type, climate type, soil pH, soil total carbon (TC), soil nitrogen (N), soil phosphorus (P), and soil carbon-to-nitrogen ratio (C/N) of each experimental site, as well as the lignin-to-nitrogen ratio (lignin/N), lignin, hemicellulose, cellulose, carbon-to-nitrogen ratio (C/N), carbon-to-phosphorus ratio (C/P), nitrogen-to-phosphorus ratio (N/P), TC, TN, and P contents of litter. Annual mean precipitation (MAP) and annual mean temperature (MAT) are shown in Figure 1.

### 2.2. Statistical Analyses

To quantify the home-field advantage in litter decomposition, this study adopted the natural logarithm of the ratio of means (*LnRR*) as the effect size metric. *LnRR* is defined as the natural logarithm of the ratio between the mean of the treatment group (home) and that of the control group (away), and its calculation formula and variance (*V_i_*) are as follows:

(1) $$LnRR=\ln(\bar{X_{H}}/\bar{X_{A}})$$

(2) $$V_{i}=\frac{S_{H}^{2}}{n_{H}\bar{X_{H}^{2}}}+\frac{S_{A}^{2}}{n_{A}\bar{X_{A}^{2}}}$$

where $\bar{X_{H}}$ and $\bar{X_{A}}$ represent the mean litter decomposition rates under home and away conditions, respectively; $S_{H}^{2}$ and $S_{A}^{2}$ represent the standard deviations of home and away conditions, respectively; and $n_{H}$ and $n_{A}$ represent the sample sizes for home and away conditions, respectively. *LnRR* > 0 indicates the presence of a home-field advantage (faster decomposition at home), *LnRR* < 0 indicates a home-field disadvantage, and *LnRR* = 0 indicates no difference. All calculations were performed using the metafor package in R (escalc function (measure = “ROM”)).

A random effects model was used to analyze the data, and the total variance of a single study case ($V_{i}^{*}$) was calculated as follows:

(3) $$V_{i}^{*}=V_{i}+\tau^{2}$$

where $\tau^{2}$ is the inter-study variance caused by different study cases.

The weight of a single study case ($w_{i}^{*}$) was calculated as follows:

(4) $$w_{i}^{*}=1/V_{i}^{*}$$

The overall mean effect size (LnRR) was back-transformed to calculate the percent difference between home and away sites as follows:

(5) $$P=(e^{\bar{LnRR}}-1)\times 100\%$$

where $e^{\bar{LnRR}}$ is the geometric mean ratio of mass loss (or decomposition rate) at home versus away sites, and P represents the percentage increase in decomposition-related metrics at home relative to away.

The cumulative effect size ($\bar{y}$) was calculated as follows, where *k* is the number of studies:

(6) $$\bar{y}=\frac{\sum_{i=1}^{k}w_{i}^{*}y_{i}}{\sum_{i=1}^{k}w_{i}^{*}}$$

The overall standard error (*SE*) was calculated as follows:

(7) $$SE=\sqrt{\frac{1}{\sum_{i=1w_{i}^{*}}^{k}}}$$

The confidence interval (CI, 95%) of the cumulative effect size was calculated as follows:

(8) $$CI=\bar{y}\pm 1.96\times SE$$

The effect of a treatment was deemed significant if the 95% CI did not overlap with zero. It indicated a positive effect when it was greater than zero.

We used R 4.5.1 with the “metafor” [41], “ggplot2” [42], “glmulti” [43], and “lavaan” [44] packages to perform the statistical analyses and generate the graphical presentations. First, we calculated the cumulative effect size by response ratio and identified the overall heterogeneity of the effect size by the random effect model with a 95% CI. For the estimation of the between-study variance (*τ*^2^) in the random-effects model, we employed the restricted maximum likelihood (REML) method, as it is recommended for continuous outcome data due to its favorable balance of bias and efficiency compared to alternatives such as the DerSimonian and Laird estimator [45]. If the heterogeneity was very strong (*Q_t_* is high, and *p* < 0.05), moderators were introduced into the model to explain it. We used a mixed-effects model to analyze the influence of the other variables on the effect size. For the categorical variables, the climate type (arid/semi-arid climates, cold/highland climates, subtropical climates, temperate climates, and tropical climates, zoned by the precipitation and temperature of the study site) and vegetation type (conifers, evergreen broadleaved, deciduous broadleaved grass, and shrub, categorized based on their morphological characteristics and representing different stages of ecosystem succession) were taken into account. Regarding continuous variables, the soil factors (pH, TC, N, P, C/N), litter factors (TC, TN, P, C/N, C/P, N/P lignin/N, lignin, hemicellulose, cellulose), and environmental factors (MAP, MAT, and altitude) were introduced into the analyses. To further evaluate the relative importance of each predictor variable for HFA, we also performed a random forest analysis using the randomForest package in R. A total of 15 predictor variables were included: altitude, MAP, MAT, soil pH, soil TC, soil N, soil C/N, litter TC, litter TN, litter P, litter C/N, litter lignin/N, litter lignin, vegetation type, and climate type. The response variable was the effect size ($y_{i}$).

#### 2.2.1. Structural Equation Modeling

To investigate the combined effects of environmental factors (altitude, mean annual temperature, mean annual precipitation), litter chemical properties (total carbon, total nitrogen, C:N ratio, lignin content), and soil pH on the effect size ($y_{i}$) across five vegetation types (evergreen broadleaved forest, coniferous forest, deciduous broadleaved forest, shrubland, and grassland), we performed path analysis using covariance-based structural equation modeling (SEM). SEM allows simultaneous examination of direct and indirect effects among variables, reveals causal relationships among environmental factors, litter/soil properties, and effect size, and evaluates the overall model fit. All structural equation models were constructed and fitted using the lavaan package in R software (version 4.5.1).

Prior to SEM construction, the missing data pattern of all variables was evaluated. Several variables exhibited high missing data rates (>50%). To ensure model stability and avoid bias caused by missing value imputation, variables with a missing data rate exceeding 50% were excluded from the model. Remaining missing values were handled using full-information maximum likelihood (FIML). The advantage of this approach lies in the fact that FIML does not impute any missing data; rather, it estimates parameters directly using all the information that is already contained in the incomplete data set. The FIML approach was outlined by Hartley and Hocking [46].

Notably, the statistical principle of FIML requires model fitting based on a single global likelihood function, which is satisfied only within the conventional SEM framework. Therefore, although piecewise SEM is flexible in handling complex data structures and non-normal distributions, we adopted traditional SEM coupled with FIML to maximize sample retention and ensure estimation accuracy.

Before modeling, raw data were scaled to bring variables with different units to comparable magnitudes, thereby preventing unstable model estimation caused by excessive variance differences. Model optimization was performed by sequentially removing non-significant paths (*p* > 0.05) and adding ecologically reasonable paths, while ensuring model degrees of freedom (df > 0) to satisfy model identification. Finally, the best-fitting model with ecologically sound interpretation was selected as the final model for each vegetation type.

#### 2.2.2. Evaluating Publication Bias

We carried out the analysis of publication bias using a fail-safe number [47]. The Rosenthal fail-safe number (*N*) quantifies publication bias in meta-analyses and indicates the number of missing studies needed to nullify the observed effect. The critical value is calculated as

N=5k+10

where *k* is the number of studies. A larger *N* indicates greater robustness of meta-analytic results against publication bias, while a smaller *N* suggests the results are vulnerable to such bias.

The analysis of publication bias showed that the Rosenthal fail safe number was 422,636, which was larger than 520 (the critical value of the fail-safe number calculated by 5*k* + 10, where *k* is the observation case number). This result suggested that there was no publication bias in the data.

## 3. Results

We ultimately identified 102 published studies that investigated litter decomposition through reciprocal transplant experiments, where litter was placed in both home and away sites. These studies covered five different climate types, five different vegetation types, and other moderators of the home advantage effect magnitude, from which we extracted 1410 study cases.

### 3.1. The Overall Effect Size of the Home-Field Effect

Overall, litter mass loss was 5.9% higher at home than away, and this difference was statistically significant (*LnRR* = 0.0576, 95% CI = 4.33 to 7.56, *p* < 0.05).

**Figure 2:**
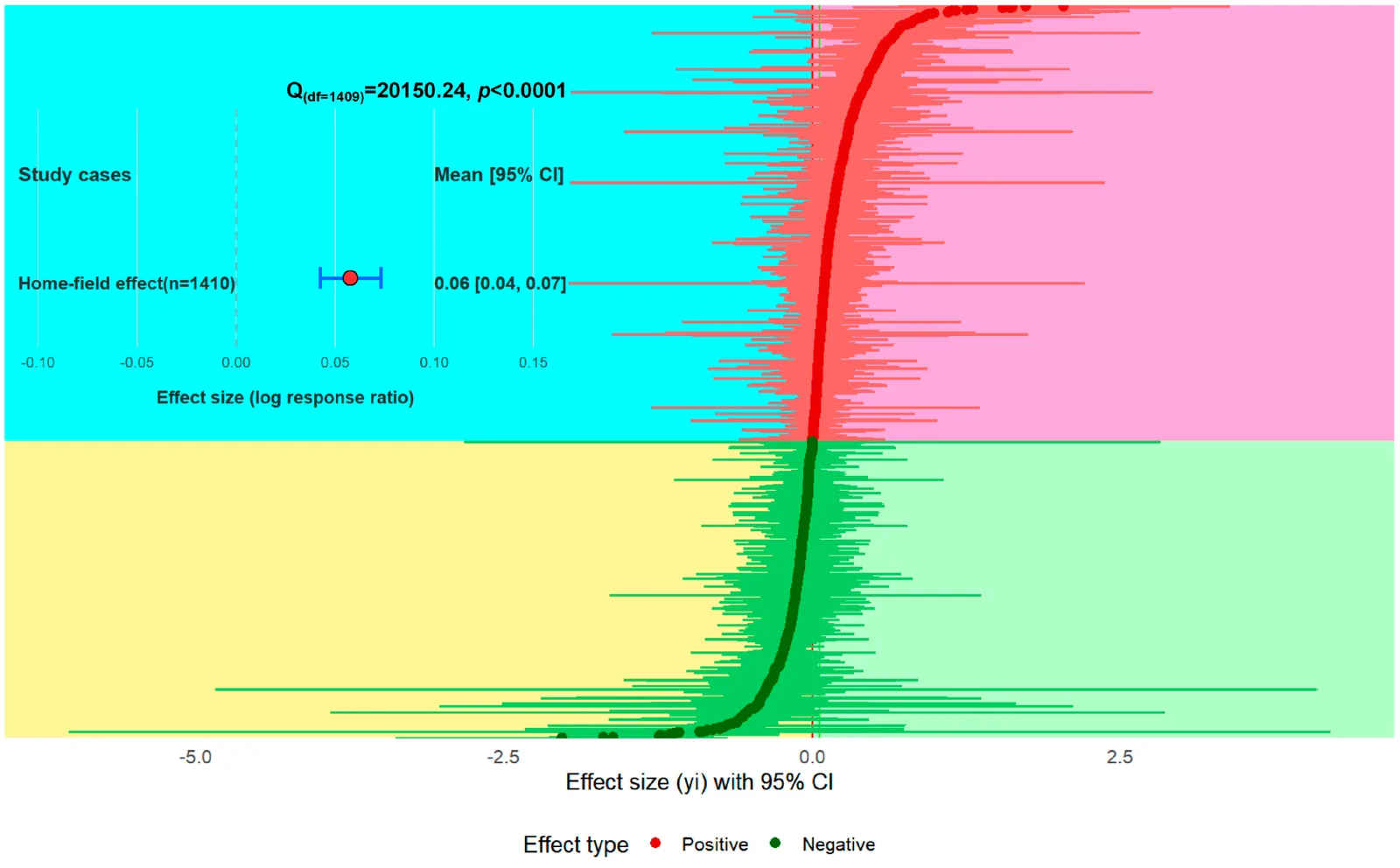
The overall effect size of the home-field effect.

Based on the random-effects model and 95% confidence intervals, the cumulative effect size for the home-field effect showed significant heterogeneity (Q_(df=1409)_ = 20,150.24, *p* < 0.001) (Figure 2). Thus, to further explain this heterogeneity, it was necessary to introduce other moderators into the random-effects model.

### 3.2. Subgroup Analysis Results: Influence of Categorical Variables on Effect Size

#### 3.2.1. Climate Type

**Figure 3:**
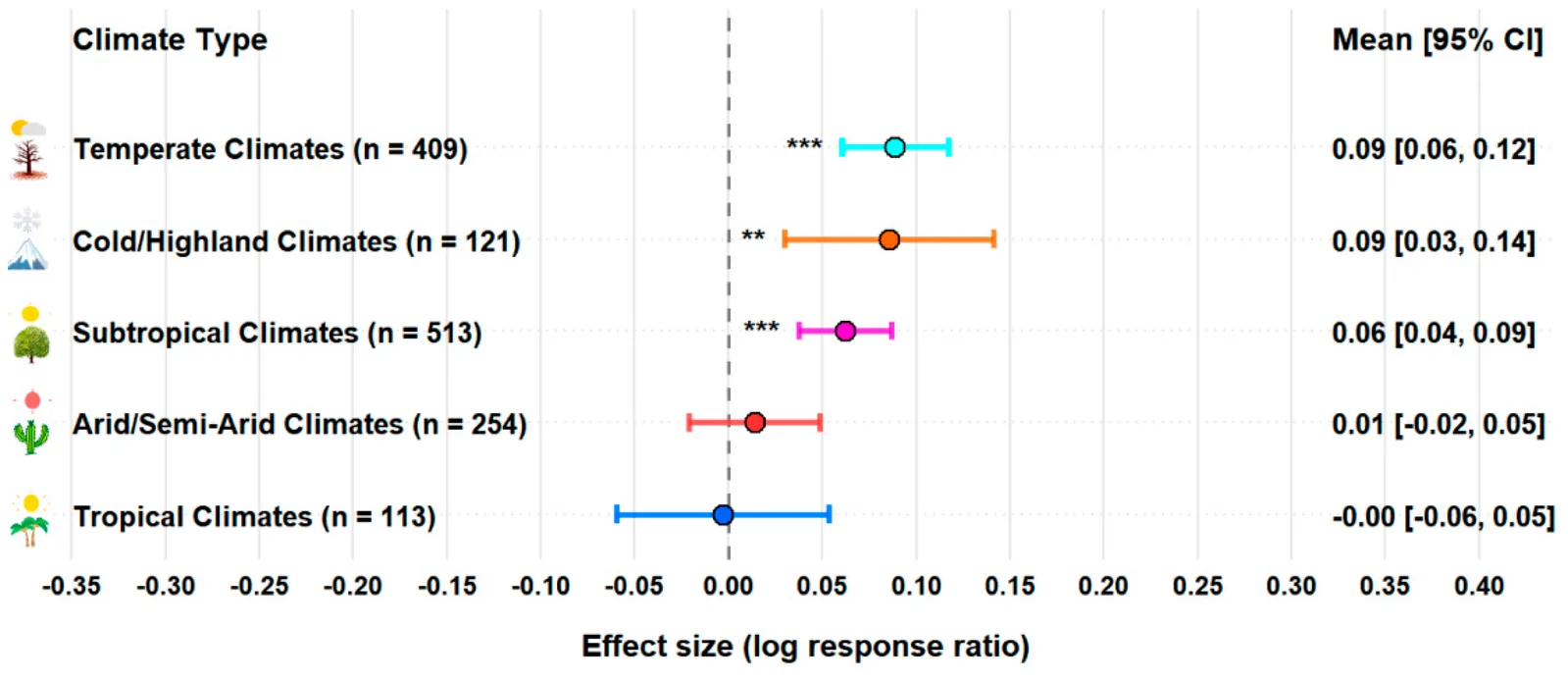
The influence of different climate types on effect size. ** *p* < 0.01 and *** *p* < 0.001 indicate significant differences between climate types.

Given that climate is a key driver of ecological processes worldwide, we included climate type as a categorical variable to examine how home-field effects vary across climatic regions (Figure 3). Significant positive home-field advantages were observed in temperate (effect size = 0.09, 95% CI: 0.06 to 0.12, *p* < 0.001, I^2^ = 93.5%), cold/highland (effect size = 0.09, 95% CI: 0.03 to 0.14, *p* < 0.01, I^2^ = 65.5%), and subtropical climates (effect size = 0.06, 95% CI: 0.04 to 0.09, *p* < 0.001, I^2^ = 96.5%). In contrast, arid/semi-arid (effect size = 0.01, 95% CI: −0.02 to 0.05, *p* > 0.05, I^2^ = 90.8%) and tropical climates (effect size = 0.00, 95% CI: −0.06 to 0.05, *p* > 0.05, I^2^ = 98.3%) showed no significant home-field effect.

#### 3.2.2. Vegetation Type

**Figure 4:**
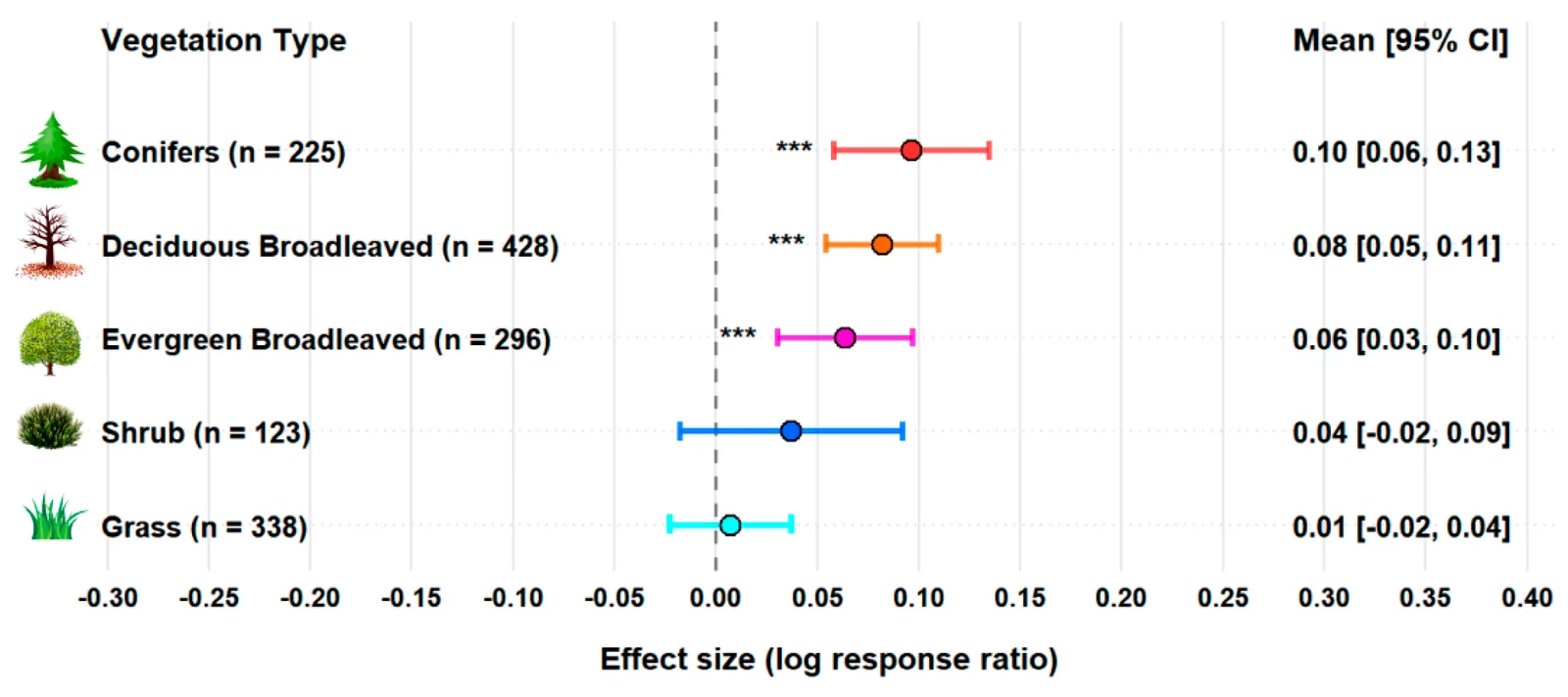
The influence of different vegetation types on effect size. *** *p* < 0.001 indicates significant differences between vegetation types.

Vegetation type is a fundamental determinant of ecosystem processes and functions. To evaluate its role in shaping home-field effects, we therefore included it as a categorical variable in the analysis (Figure 4). A significant positive home-field effect was detected in three vegetation types: conifers (effect size = 0.10, 95% CI: 0.06 to 0.13, *p* < 0.001, I^2^ = 93.5%), deciduous broadleaved (effect size = 0.08, 95% CI: 0.05 to 0.11, *p* < 0.001, I^2^ = 96.6%), and evergreen broadleaved forests (effect size = 0.06, 95% CI: 0.03 to 0.10, *p* < 0.001, I^2^ = 97.5%). By contrast, no significant effect was observed for shrub (effect size = 0.04, 95% CI: −0.02 to 0.09, *p* > 0.05, I^2^ = 78.5%) or grass (effect size = 0.01, 95% CI: −0.02 to 0.04, *p* > 0.05, I^2^ = 90.3%).

Notably, the effect size exhibited a clear increasing gradient across vegetation types, from grass (0.01) to conifers (0.10), suggesting that home-field advantage strengthens along a gradient from grasslands to coniferous forests.

### 3.3. Meta-Regression Results: The Influence of Continuous Variables on Effect Size

#### 3.3.1. Altitude, MAP, MAT

**Figure 5:**
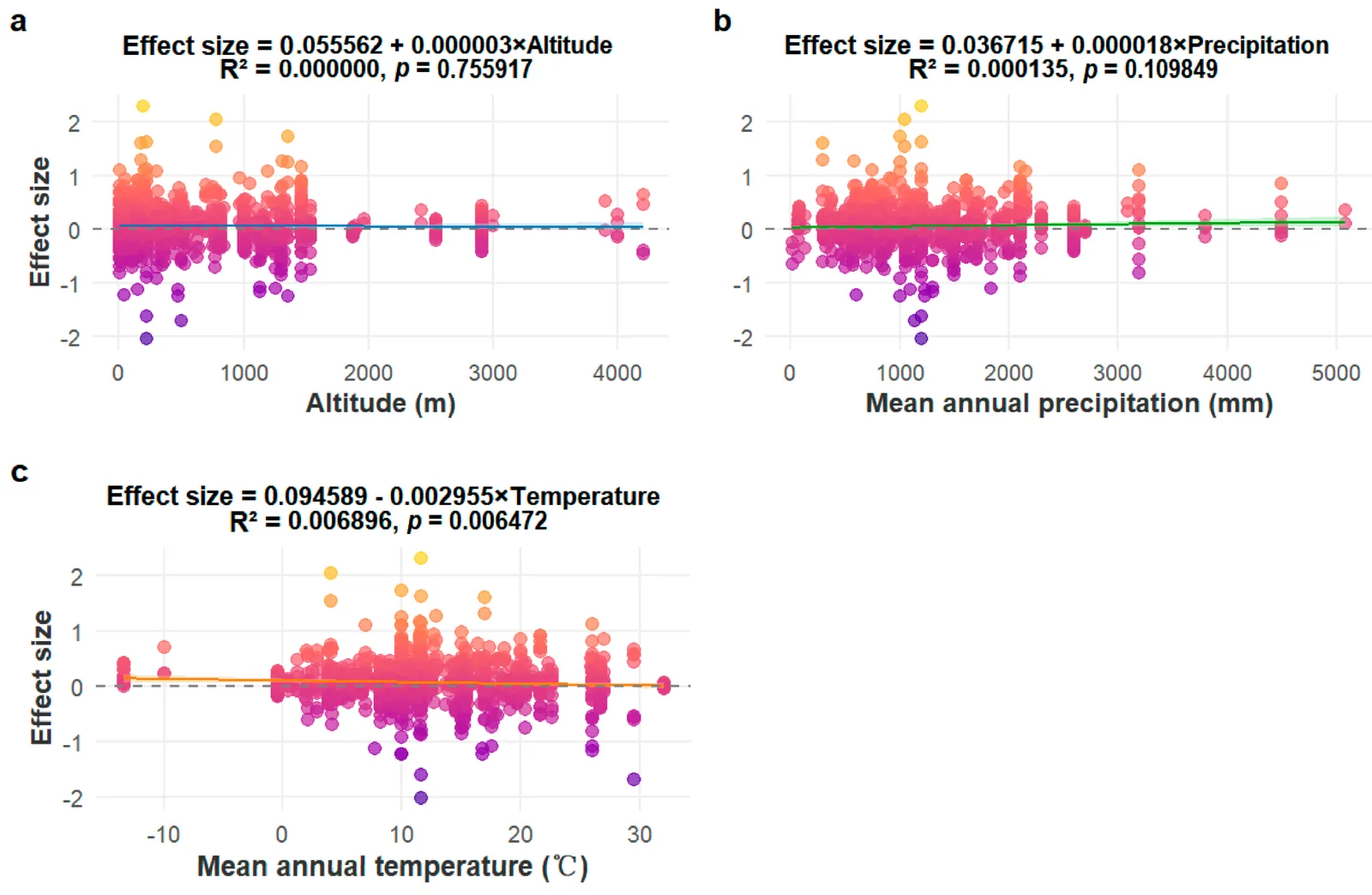
Relationships between the effect size of different environment factors ((**a**) altitude; (**b**) MAP; and (**c**) MAT).

Meta-regression analysis showed no significant linear relationship between altitude and the effect size of the home-field advantage (HFA) in litter decomposition (Figure 5a). The model had an extremely low coefficient of determination (R^2^ < 0.001) and a non-significant *p*-value (*p* = 0.755917), indicating that altitude could not explain substantial variation in HFA effect sizes within the range of 0–4000 m in this study. The wide dispersion of data points around the regression line further confirms that altitude is not a major factor driving HFA heterogeneity in our dataset, suggesting that other environmental or biological factors may obscure the direct influence of altitudinal gradients on decomposition specificity.

The linear regression between mean annual precipitation (MAP) and effect size showed a weak, non-significant positive correlation (Figure 5b). The model had an extremely low coefficient of determination (R^2^ = 0.000135) and a non-significant *p*-value (*p* = 0.109849). Although the slope indicated a slight increase in effect size with increasing precipitation (0–5000 mm), the non-significant *p*-value suggests that MAP alone cannot reliably predict variation in HFA. This implies that the relationship between MAP and HFA may be context-dependent or interact with other factors (e.g., temperature), rather than being driven by a simple linear mechanism.

A significant negative correlation was observed between mean annual temperature (MAT) and effect size (Figure 5c). The model had a coefficient of determination of R^2^ = 0.006896 and a significant *p*-value (*p* < 0.01), indicating that, for every 1 °C increase in MAT, the effect size decreased by approximately 0.002955 units. This suggests that rising temperatures may weaken the strength of HFA, potentially because higher temperatures accelerate overall litter decomposition rates, thereby reducing the relative advantage of native decomposer communities over foreign substrates.

#### 3.3.2. Soil Properties: Soil TC, Soil N, Soil P, Soil C/N, and Soil pH

**Figure 6:**
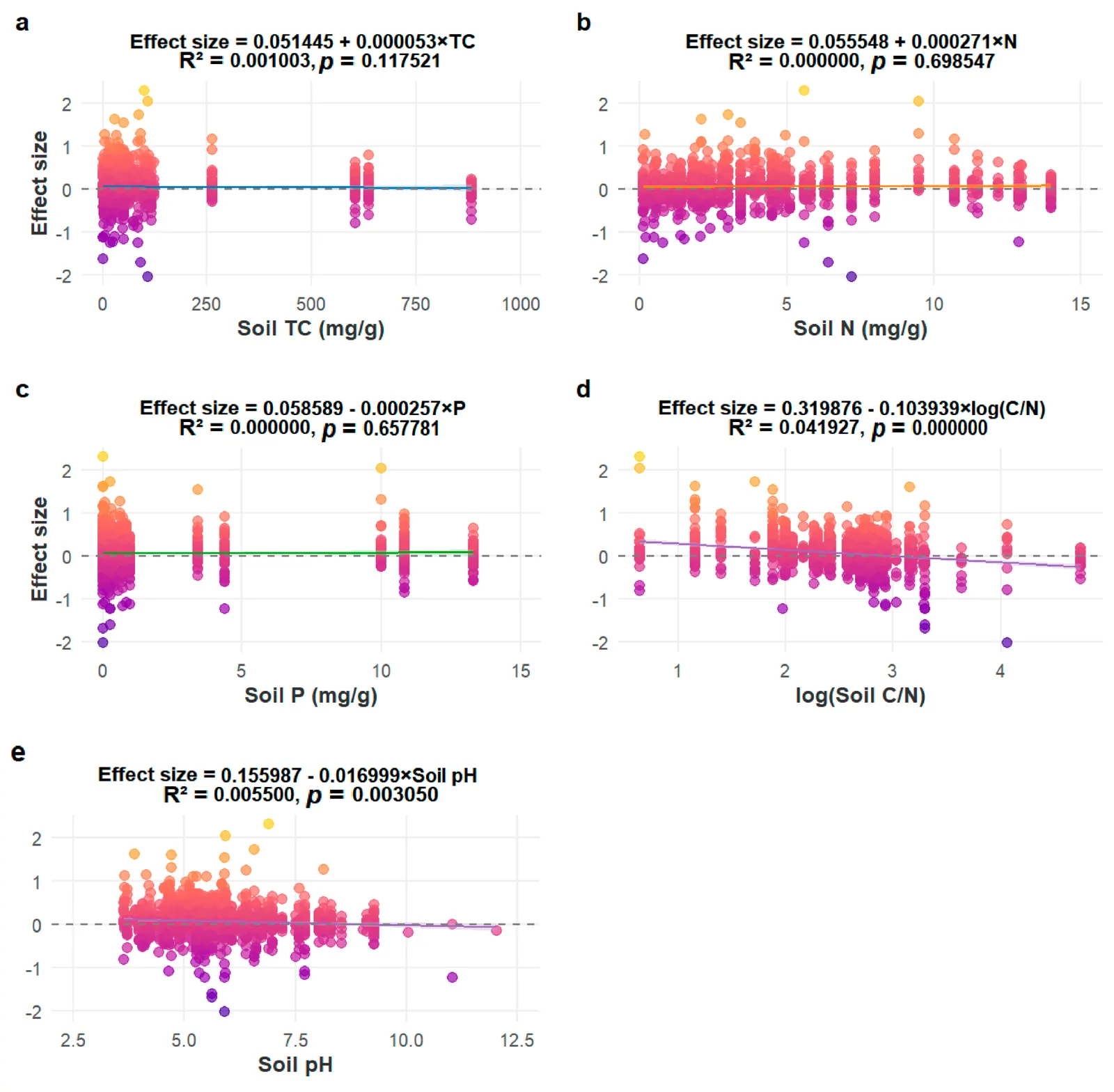
Relationships between effect size and soil factors ((**a**) soil total carbon (TC); (**b**) soil nitrogen (N); (**c**) soil phosphorus (P); (**d**) logarithm (base 10) of soil carbon-to-nitrogen ratio (C/N); and (**e**) soil pH).

Positive but statistically non-significant correlations with effect size were observed for both soil total carbon (TC) and soil nitrogen (N) in meta-regression analyses (Figure 6a,b). Both models exhibited extremely low coefficients of determination (TC: R^2^ = 0.001003, *p* = 0.117521; N: R^2^ < 0.001, *p* = 0.698547).

A negative correlation was observed between soil P and effect size, which was not statistically significant (Figure 6c). The model had a coefficient of determination of zero (R^2^ = 0.000000) and a non-significant *p*-value (*p* = 0.657781).

A significant negative correlation was observed between soil C/N ratio and effect size (Figure 6d). The model had a coefficient of determination of R^2^ = 0.041927 and a highly significant *p*-value (*p* < 0.001), indicating that, for every one-unit increase in log (C/N), the effect size decreased by approximately 0.104 units.

Soil pH (aqueous extraction) also showed a significant correlation with effect size (Figure 6e). The model had a coefficient of determination of R^2^ = 0.0055 and a significant *p*-value (*p* < 0.01), indicating that, for every one-unit increase in soil pH, the effect size decreased by approximately 0.017 units. This significant negative trend suggests that more acidic soils tend to strengthen the HFA effect, whereas alkaline environments weaken decomposition specificity.

#### 3.3.3. Litter Properties: Litter TC, Litter TN, Litter P, Litter C/N, Litter C/P, and Litter N/P

**Figure 7:**
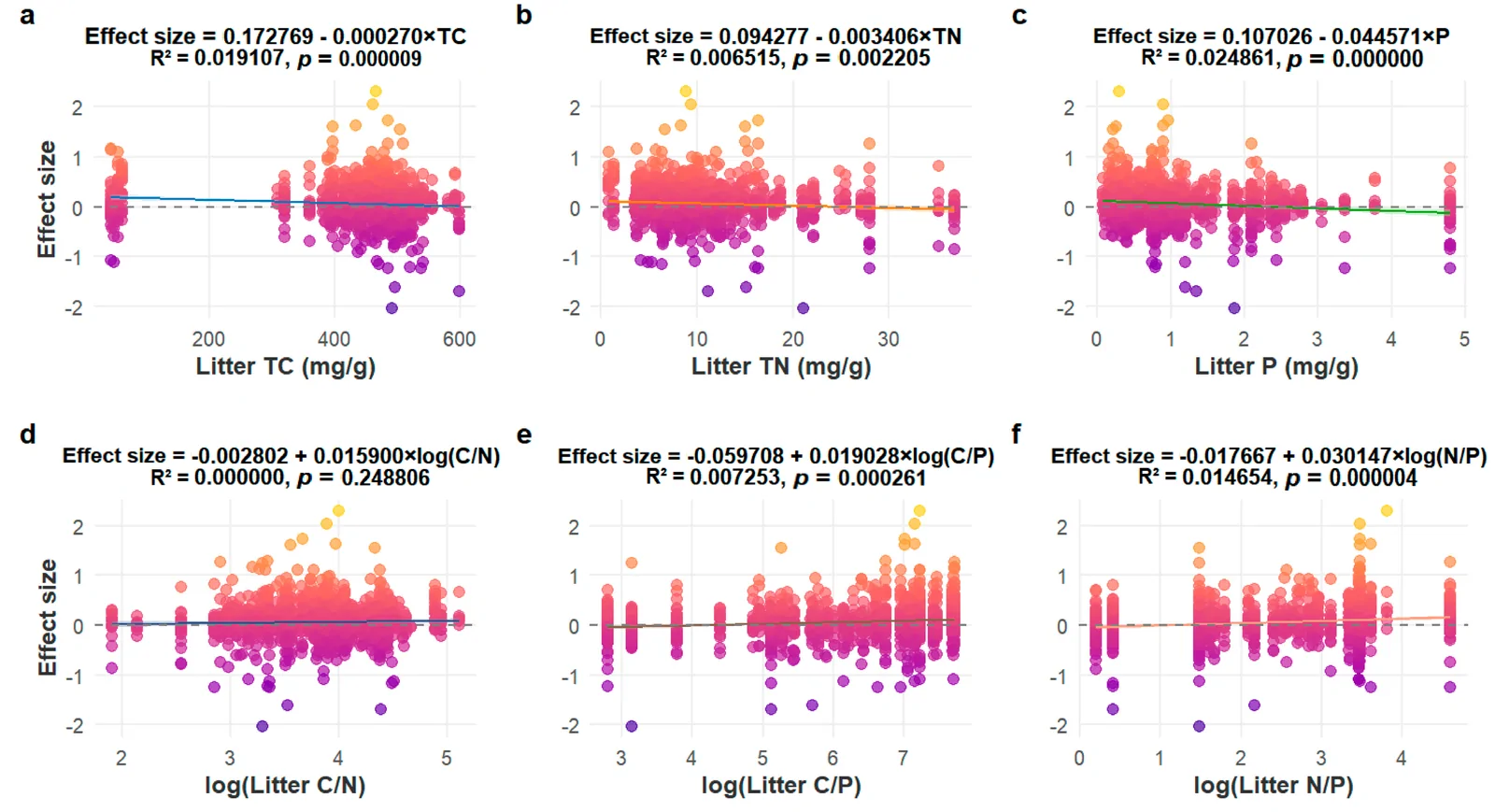
Relationships between effect size and litter factors ((**a**) litter total carbon (TC); (**b**) litter total nitrogen (TN); (**c**) litter phosphorus (P); (**d**) logarithm (base 10) of litter carbon-to-nitrogen ratio (C/N); (**e**) logarithm (base 10) of litter carbon-to-phosphorus ratio (C/P); and (**f**) logarithm (base 10) of litter nitrogen-to-phosphorus ratio (N/P)).

Significant negative correlations with effect size were found for litter total carbon (TC), total nitrogen (TN), and phosphorus (P) (Figure 7a–c), with the models explaining 1.91% (*p* < 0.001), 0.65% (*p* = 0.002205), and 2.49% (*p* < 0.001) of the variance, respectively. These results indicate that higher litter nutrient content consistently weakens the home-field advantage in decomposition.

Positive correlations with effect size were found for litter C/N, C/P, and N/P ratios (Figure 7d–f). The C/N ratio showed a non-significant trend, while both C/P (R^2^ = 0.007253, *p* < 0.001) and N/P (R^2^ = 0.014654, *p* < 0.001) were significantly positively correlated, indicating that higher litter stoichiometric ratios are associated with a stronger home-field advantage.

#### 3.3.4. Litter Properties: Litter Lignin/N, Litter Lignin, Litter Hemicellulose, and Litter Cellulose

**Figure 8:**
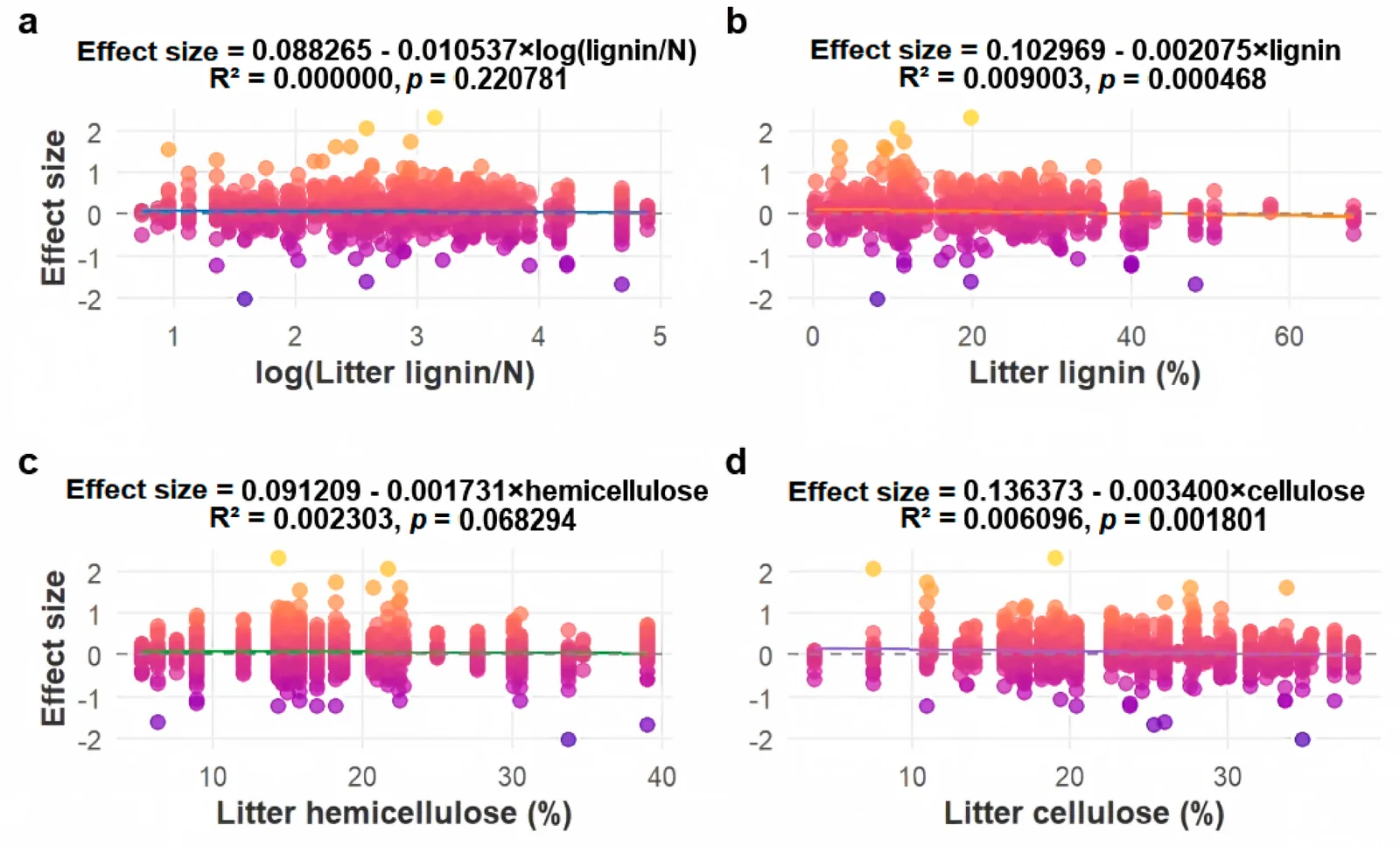
Relationships between the effect size of different litter factors ((**a**) logarithm (base 10) of litter lignin-to-nitrogen ratio (lignin/N); (**b**) percentage of litter lignin; (**c**) percentage of hemicellulose; and (**d**) percentage of litter cellulose).

Negative correlations with effect size were found for all four litter components—lignin/N, lignin, hemicellulose, and cellulose (Figure 8a–d). Significant negative associations were detected for lignin (R^2^ = 0.009003, *p* < 0.001) and cellulose (R^2^ = 0.006096, *p* < 0.001), while lignin/N and hemicellulose showed non-significant negative trends (lignin/N: R^2^ < 0.001; hemicellulose: R^2^ = 0.002303).

### 3.4. The Importance of Environmental Factors to the Effect Size

**Figure 9:**
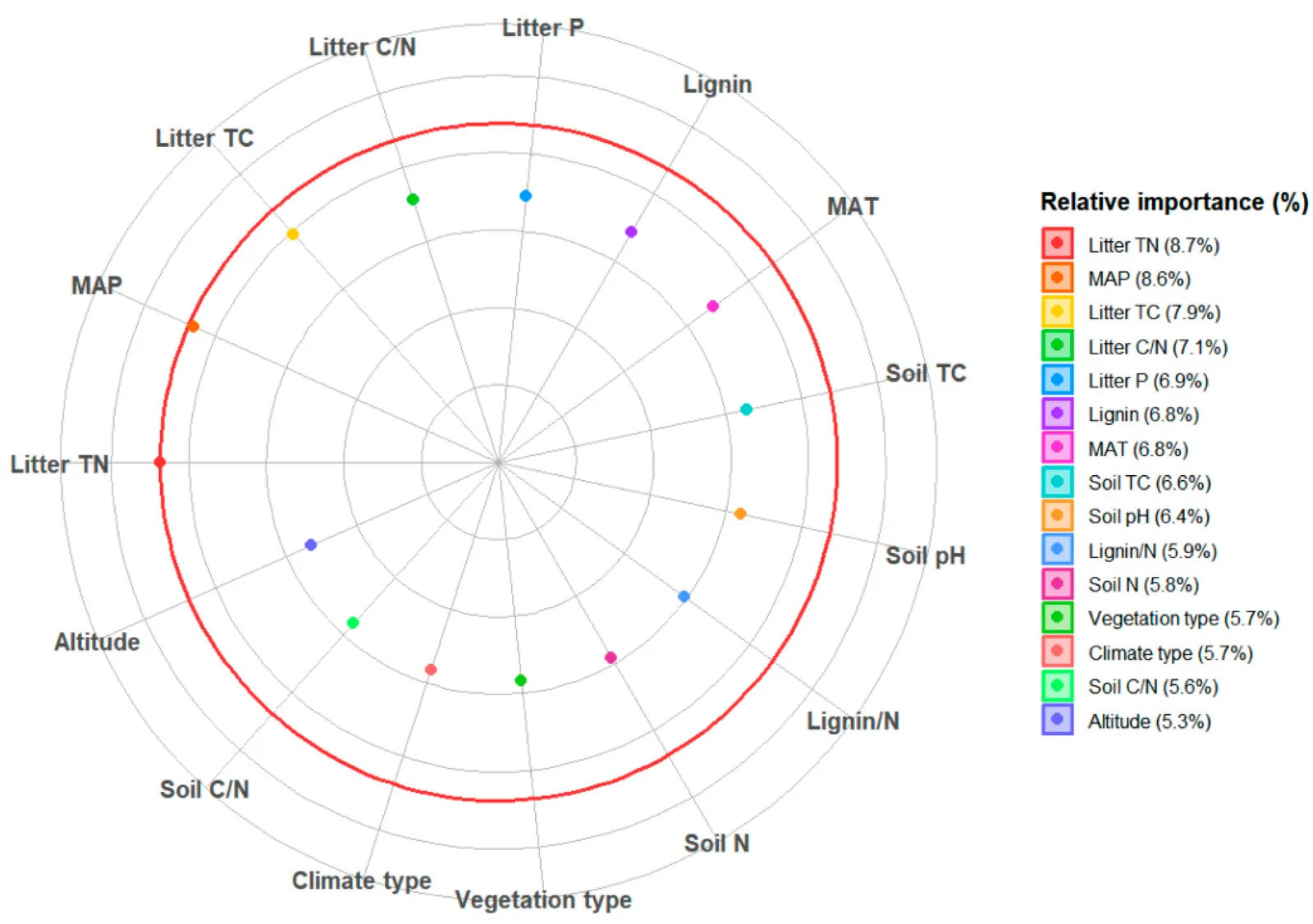
Relative importance of environmental factors on effect size based on the importance analysis of the random forest model.

Figure 9 presents a relative importance radar plot illustrating the relative contribution of various environmental factors to the effect size of home-field advantage (relative importance ranges from 0% to 100%, with higher values indicating a stronger influence). Among all factors, litter total nitrogen (Litter TN) exhibited the highest relative importance (8.7%), serving as the dominant factor driving the effect size. Mean annual precipitation (MAP, 8.6%) and litter total carbon (Litter TC, 7.9%) followed as co-dominant factors, ranking second and third, respectively. Factors including Litter C/N (7.1%), Litter P (6.9%), Litter lignin (6.8%), mean annual temperature (MAT, 6.8%), soil total carbon (Soil TC, 6.6%), and soil pH (6.4%) showed moderate importance, exerting a weak but non-negligible influence. In contrast, factors such as soil C/N (5.6%) and altitude (5.3%) were located close to the center of the radar plot, indicating their negligible contribution to the effect size. These results collectively suggest that litter quality (e.g., Litter TN, Litter TC) and climatic factors (e.g., mean annual precipitation) are the primary drivers of the home-field advantage effect size, whereas soil properties play a minor role.

### 3.5. Evaluating Publication Bias

**Figure 10:**
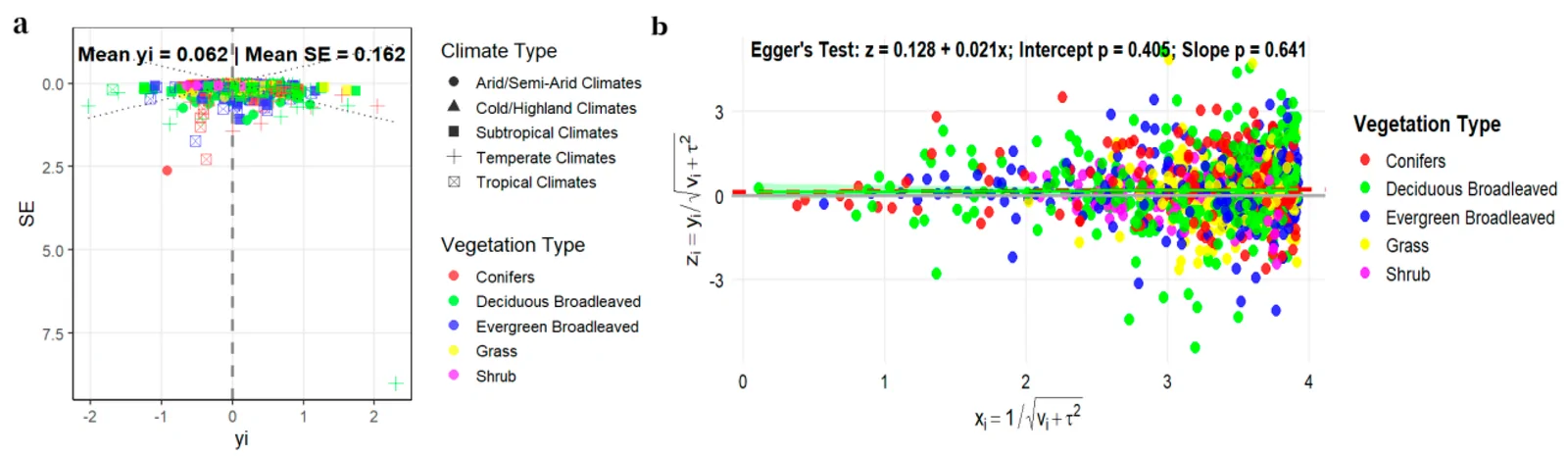
Funnel plot analysis. (**a**) Funnel plot analysis grouped by vegetation types and climatic types. (**b**) Funnel plot analysis grouped by vegetation types with Egger’s test.

The results show that the mean effect size is 0.062, with a corresponding mean standard error of 0.162; most study points are concentrated around the mean effect size, with a relatively compact distribution of standard errors. The plot exhibits a nearly symmetric funnel shape overall, with no obvious one-sided point absence or deviation. Egger’s linear regression test (Figure 10b) further quantitatively verified the symmetry of the funnel plot: the *p*-value for the intercept term is 0.405, and that for the slope term is 0.641, both exceeding the 0.05 significance level. This indicates that the asymmetry of the funnel plot is not statistically significant. In conclusion, there is no significant publication bias in the data of this study.

To assess the publication bias of the data in this study, a funnel plot combined with Egger’s linear regression test was employed. Figure 10 shows a funnel plot of effect size (yi) versus standard error (SE).

### 3.6. Structural Equation Model Analysis

**Figure 11:**
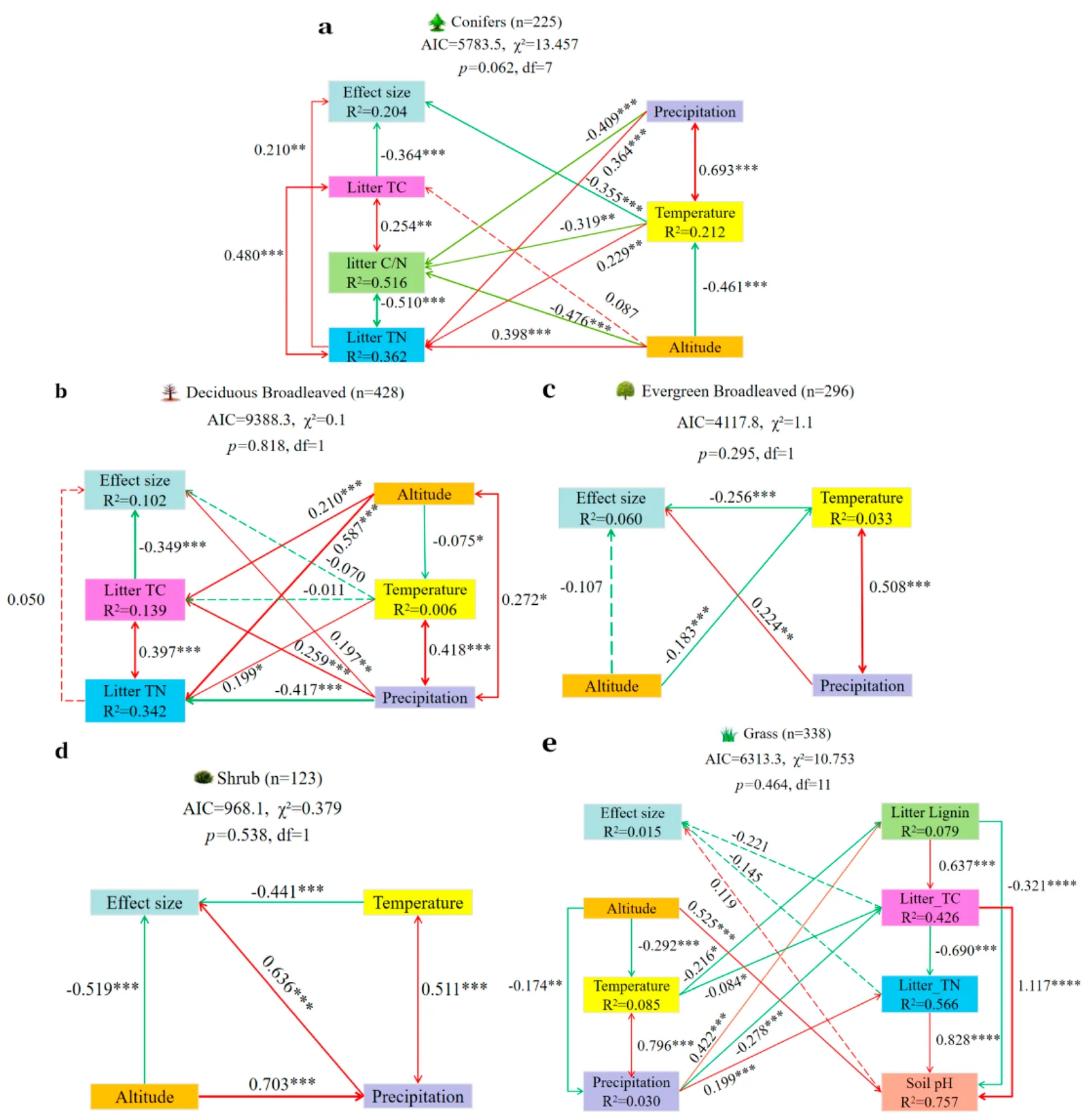
Structural equation model of effect size interactions with altitude, temperature (mean annual temperature), precipitation (mean annual precipitation), Litter TC, Litter C/N, Litter TN, Litter Lignin, and soil pH. Solid arrows indicate notable effect sizes, *p* < 0.05, dashed arrows indicate *p* > 0.05, and the thickness of the arrow represents the strength of the relationship. Red and green lines indicate positive and negative relationships, respectively. (**a**) Conifers; (**b**) deciduous broadleaved; (**c**) evergreen broadleaved; (**d**) shrub; (**e**) grass. Significance levels for each predictor are denoted as * *p* < 0.05, ** *p* < 0.01, *** *p* < 0.001.

In order to further explore the causal relationship between variables and the relative importance of effect size, structural equation models were fitted for five vegetation types. All five models demonstrated strong goodness-of-fit (Figure 11).

For coniferous forests, litter quality and climate jointly regulated the strength of the home-field advantage. Litter total nitrogen (β = 0.210, *p* < 0.01) exerted a significant positive influence on effect size, contrary to the pattern observed in the univariate linear regression. Litter total carbon (β = −0.364, *p* < 0.001) and temperature (β = −0.355, *p* < 0.001) showed significant negative effects on effect size, consistent with the univariate results (Figure 11a).

Regarding evergreen broadleaved forests, temperature emerged as a significant negative predictor of effect size (β = −0.256, *p* < 0.001), consistent with the univariate analysis. Altitude also had a negative effect (β = −0.107), though it was not significant. In contrast to temperature and altitude, precipitation exhibited a significant positive effect (β = 0.224, *p* < 0.01) (Figure 11b).

For deciduous broadleaved forests, precipitation had a significant positive effect on effect size (β = 0.197, *p* < 0.01). Litter total nitrogen (β = 0.050) exhibited a positive but non-significant influence, which contrasted with the univariate analysis. Litter total carbon (β = −0.349, *p* < 0.001) showed a significant negative effect, aligning with the univariate findings. Temperature (β = −0.070) also exerted a negative but non-significant influence (Figure 11c).

For shrublands, precipitation had a significant positive effect on effect size (β = 0.636, *p* < 0.01). Both altitude and temperature exhibited significant negative effects (β = −0.519, *p* < 0.01; β = −0.441, *p* < 0.001, respectively) (Figure 11d).

For grasslands, litter total carbon (β = −0.221) and litter total nitrogen (β = −0.145, *p* < 0.01) showed negative but non-significant effects on effect size. Soil pH also had a non-significant effect, though its influence was positive—contrary to the univariate pattern (Figure 11e).

## 4. Discussion

Our meta-analysis provides the first comprehensive global assessment of biogeographical differentiation in the home-field advantage (HFA) effect across terrestrial ecosystems.

By integrating 1410 study cases from 102 publicly available papers worldwide, this meta-analysis systematically evaluated the global pattern, biogeographical differentiation, and drivers of the HFA effect in litter decomposition across habitats. The results confirm the ubiquity of the HFA effect globally and reveal that its intensity is governed by the synergistic regulation of climate, vegetation, litter chemistry, and soil properties, providing a novel theoretical foundation for understanding spatial heterogeneity in terrestrial carbon and nutrient cycling. This biogeographical differentiation challenges the traditional assumption that HFA operates uniformly across ecosystems and underscores the need for climate- and vegetation-specific decomposition models.

### 4.1. Overall Pattern and Biogeographic Differentiation

Globally, we found a significantly positive HFA effect overall, with decomposition metrics (e.g., mass loss, decomposition rate) being 5.9% higher at home sites than at away sites (Figure 2)—a magnitude that is broadly consistent with previous regional-scale syntheses [12,13,27].

Litter decomposition plays a key role in ecosystem service provision by converting plant material into easily accessible inorganic components that are used by soil heterotrophs and plants [48]. By producing litters with differing chemical compositions, plants influence the activity and composition of the organisms involved in decomposition, which in turn affects carbon and nitrogen cycling in soils [49]. Accordingly, decomposers differ in their consumption and decomposition capacities for different litters due to feeding preferences and physiological traits (e.g., enzyme production capacity) [37,50,51].

The drivers of litter decomposition include three aspects: physicochemical environment (e.g., temperature, humidity, light), litter quality (e.g., lignin/N), and organisms involved in the decomposition process (e.g., bacteria, fungi, and invertebrates) [52]. Among these, the activity of soil biota is considered to be controlled by temperature, humidity, and litter quality, so the role of soil biota has been largely overlooked [53]. The specialization of soil biota in decomposing litter from the same habitat is considered to be the fundamental cause of the HFA effect [12,54]. Soil biota (including microorganisms, protozoa, nematodes, microarthropods, and macrofauna) regulate litter decomposition while simultaneously absorbing energy and nutrients, creating competition among different soil organisms. This leads to the specialization of soil microorganisms in decomposing litter from plant species in the same habitat, generating the HFA effect [12]. The specialization of soil microorganisms in litter decomposition can be demonstrated in various ways. Soil organisms produce specific enzymes to decompose different litter substrates, but a single species or group often cannot produce enzymes to decompose certain substrates [12]. For example, lignin-degrading fungi are among the few microorganisms that can effectively decompose lignin, and they are therefore more abundant in soils of forests that produce high-lignin litter [55]. Li et al. [56] reported that HFA driven by bacteria and fungi may be attributed to specific microbial taxa, such as Xanthomonadales and Cutaneotrichosporon. Similarly, Veen et al. [29] found that overall differences in fungal community composition between home and away sites were not significantly related to HFA, except for specific fungal taxa such as Sordariomycetes, which showed significant positive correlations with HFA. Moreover, we found that microbial community dissimilarity between home and away sites was closely linked to HFA for mass loss [29,57,58].

### 4.2. Regulation by Climate Type

Despite this significant overall effect, pronounced biogeographical differentiation was observed. Specifically, the HFA effect was significant in temperate, cold/highland, and subtropical zones, but non-significant in tropical and arid/semi-arid zones. This is likely due to differences in soil microbial communities and distinct climatic conditions [59,60].

Our study shows that plant litter decomposes faster in temperate and subtropical regions (Figure 3), which is consistent with previous findings. As reported by Wan et al. [61], a 4-year reciprocal transplant experiment found that, in mild years (i.e., 2016 and 2018), both litter origin and litter degradability effects were highly significant. Lin et al. [62] found that, in a subtropical reciprocal transplant experiment with three forest types (broadleaf, coniferous, and bamboo forests), mass loss of broadleaf and bamboo litter was greater at home sites than at away sites. This was mainly driven by the significant effect of incubation site on fungi colonizing broadleaf and bamboo litter.

However, in arid and semi-arid regions, the absence of HFA effects may be attributed to the following mechanisms. On one hand, biological soil crusts may dominate the decomposition process. Miralles et al. [63] found that, in the Tabernas Desert of Spain, biological soil crusts exhibited various hydrolase enzyme activities involved in carbon (cellulase, β-glucosidase, and invertase), nitrogen (casein protease and BAA protease), and phosphorus (alkaline phosphomonoesterase) cycles, with all enzyme activities significantly higher than in bare substrate. This suggests that litter decomposition in arid zones may be primarily driven by a generalist enzymatic process associated with biological soil crusts, rather than by the specialization of specific microbial communities to local litter, thereby potentially weakening or masking the traditional HFA effect. On the other hand, extreme environmental stress may overshadow any weak local adaptation effects. Xiao et al. [64] demonstrated that drought significantly inhibited soil carbon release across global terrestrial ecosystems, with particularly strong effects in humid regions, further supporting the overriding role of water availability in regulating decomposition processes under dry conditions. In tropical rainforests, the absence of HFA may be attributed to functional redundancy among decomposer communities. Ferreira-Santos et al. [65] found that, in the Brazilian Atlantic Forest (Mata das Flores State Park), although the abundance and structure of Rubiaceae communities changed significantly after drought, their functional composition, as well as litter decomposition rates (mass loss), remained unchanged, indicating that functionally similar species can replace each other following disturbance, thereby maintaining the stability of ecosystem function. This finding directly supports the functional redundancy hypothesis, suggesting that, in species-rich tropical forests, decomposition functions can be sustained by functionally similar species even when the abundance of certain species changes. Such functional redundancy may weaken the close specialization between specific decomposers and local litter, thereby potentially suppressing the manifestation of HFA effects.

These results suggest that home-field advantage is more pronounced in temperate, cold/highland, and subtropical regions but absent in arid and tropical climates, highlighting the role of climatic context in modulating litter decomposition dynamics.

### 4.3. Regulation by Vegetation Type

Similarly, vegetation type exerted a strong moderating effect: significant HFA was confined to forest ecosystems (coniferous, deciduous broadleaved, and evergreen broadleaved forests), while shrublands and grasslands showed no significant HFA. First, forests typically have higher humidity and temperature, which enhance the metabolic activity of decomposer microbial communities and accelerate carbon decomposition [50]. Second, differences in microbial nutrient limitation between ecosystems lead to varying degrees of microbial growth constraint, thereby causing differences in decomposition rates. Forest soils with long-term organic matter accumulation maintain more stable nutrient cycling and provide a balanced and continuous nutrient supply for microorganisms [66]. Finally, differences in the functional potential of microbial decomposers are also important contributors to decomposition differences [67,68]. Forest soils with long-term exposure to complex and recalcitrant litter select for more diverse microbial communities with stronger decomposition capacities.

In contrast, the absence of significant HFA in shrubland ecosystems may be partly attributed to its temporal instability. Zheng et al. [69] found that, in an alpine shrubland, HFA effects were present during the first year of decomposition but diminished significantly in the second year. This suggests that HFA in shrublands is not a consistent phenomenon, but rather weakens over time, which is consistent with the overall non-significant HFA detected for shrublands in our meta-analysis. In grassland ecosystems, Wang et al. [70] conducted a reciprocal litter transplant experiment in a semi-arid steppe and found no evidence of HFA across different land-use types. Instead, litter decomposition was consistently faster in enclosed grasslands than in grazed or mowed grasslands, regardless of litter origin, and this pattern was primarily driven by higher soil moisture in the enclosed sites. These findings suggest that, in semi-arid grasslands, environmental factors such as soil moisture can override the effects of litter quality and microbial community specialization, thereby masking any potential HFA effect.

Notably, coniferous forests, which typically support higher mesofaunal abundance than broadleaved forests, exhibit stronger HFA effects [58]. This is consistent with our finding that significant HFA occurs only in forest ecosystems (especially coniferous forests) with stronger functional capacity, while shrublands and grasslands show no significant HFA.

Collectively, these findings indicate that climate and vegetation type jointly shape the macro-geographical distribution of HFA through their effects on microbial communities and litter–microbe interactions.

### 4.4. Direct Regulation by Environmental Factors

Integrated regression analysis and structural equation modeling (SEM) further elucidated the direct and indirect regulatory roles of environmental factors. Our results showed that MAP (8.6%) and MAT (6.8%) (variable importance values from the random forest analysis; Figure 9) both exhibited high relative importance in influencing HFA effects.

Climate and litter quality are widely recognized as major regulators of decomposition [24,71]. Our results further demonstrate that they also modulate the magnitude of the HFA effect. Specifically, MAP showed a significant positive correlation with HFA, consistent with previous studies, as precipitation can indirectly affect litter decomposition rates by regulating the activity, abundance, and diversity of soil microorganisms [72,73]. In contrast, MAT exhibited a significant negative correlation with HFA, consistent with the finding of stronger HFA effects in colder regions. This result may be attributed to the adaptive specialization of microbial communities under low-temperature conditions—in colder regions, microbial communities may develop stronger substrate preferences and enhanced capacity to decompose local litter, thereby amplifying the decomposition differences between home and away environments [72].

Although we are aware that temperature and precipitation are crucial drivers of litter decomposition at large spatial scales [50,74], we know little about the effects of varying climate over time on the consistency of HFA effects. Changes in temperature and precipitation over time could affect litter decomposition in grasslands and forest ecosystems differently, mainly because forests have a greater capacity to maintain stable and buffered environments in the face of climate extremes compared to grasslands [75,76,77]. Thus, varying climates could affect the consistency of HFA effects across ecosystems differing in vegetation type and/or soil fertility.

### 4.5. Regulation by Litter Chemical Properties

In our study, litter chemical properties consistently emerged as central drivers of HFA effect intensity, supporting our second hypothesis. Litter TN and P contents were significantly negatively correlated with HFA effect intensity, suggesting that high-quality, easily decomposable litter (e.g., high N and P content) does not require specialization of the soil decomposer community. This is consistent with previous findings. As Pugnaire et al. [78] demonstrated, at the Atlas and Sierra Nevada sites, most soil communities contained biota capable of rapidly processing litter, resulting in little or no HFA—in other words, the local soil community lacked the special ability to decompose this high-quality litter. In their experiment, the Atlas and Sierra Nevada systems produced the most decomposable litter due to the higher proportion of nutrient-rich forbs, and the proportion of this functional group in the community showed an inverse relationship with HFA.

Conversely, Litter C/N, C/P, and N/P ratios showed significant positive correlations with HFA effects. Lignin content exhibited a significant negative correlation with HFA effects. Milcu et al. [11] emphasized that litter quality (as indicated by lignin, C/N ratio, etc.) is a key factor regulating decomposition rates, particularly the HFA effect. Our study provides strong quantitative support for this conclusion in a large-scale multi-factor comparison. Our results showed that, among all environmental factors, the sum of importance values of indicators directly related to litter quality (Litter C/N, C/P, N/P, lignin) ranked at the forefront, with Litter TN ranking first, with a relative importance of 8.7%. This is consistent with the important role of soil microorganisms in nutrient cycling [79], as litter quality largely determines the substrate availability and energy supply for microbial decomposers.

Therefore, plant communities drive soil microbial community structure through litter production and quality, and such plant–soil feedback is critical for governing the co-evolution of above- and belowground communities [80,81,82]. The specialization of soil fauna also requires favorable environmental factors such as light, temperature, and humidity, which leads to variability in HFA across different systems.

Overall, our findings support that HFA is a consistent global phenomenon, with home litter decomposing faster than away litter on average. Litter chemical traits (especially C/P and N/P ratios) and certain environmental factors (MAT and soil pH) significantly modulated HFA intensity, consistent with our hypothesis that litter quality and environmental conditions jointly regulate HFA. However, the selected soil chemical indicators in this study showed limited explanatory power for HFA variation. This does not necessarily exclude an important role of soil microorganisms, but suggests that microbial mechanisms may not be fully captured by bulk soil chemical properties. These results underscore the need to consider litter quality and key environmental factors when predicting HFA patterns across broad geographic scales.

### 4.6. Limitations and Future Research

Several limitations of this study should be acknowledged. First, although our meta-analysis included 1410 observations from 102 studies, the geographic distribution of the included studies was uneven, with most studies concentrated in temperate and subtropical regions, while tropical and arid regions were underrepresented. This imbalance may limit the generalizability of our findings to these less studied ecosystems.

Second, due to data availability, our analysis relied on litter chemical traits and soil chemical properties as moderators but lacked direct measurements of microbial community composition, functional genes, or extracellular enzyme activities. Consequently, the limited explanatory power of soil properties observed in this study should be interpreted with caution, as it reflects the selected chemical indicators rather than the role of soil microorganisms themselves.

Third, substantial heterogeneity existed across the included studies, with I^2^ values ranging from 65.5% to 98.3% across different vegetation types and climate zones (all *p* < 0.001). This high heterogeneity may arise from differences in experimental design, litterbag mesh sizes, incubation durations, and site-specific conditions.

For future research, we recommend (1) more standardized and long-term reciprocal transplant experiments, particularly in underrepresented ecosystems such as tropical rainforests, arid regions, grasslands, and shrublands; (2) integration of microbial functional indicators (e.g., microbial biomass carbon/nitrogen, extracellular enzyme activities, and microbial carbon use efficiency) to better elucidate the mechanisms underlying HFA; and (3) further exploration of the interactions among litter quality, climate, and soil properties to improve predictive models of HFA patterns in the context of global environmental change.

## 5. Conclusions

This study assessed the global patterns, biogeographical differentiation, and key drivers of the home-field advantage (HFA) effect in cross-habitat litter decomposition and drew several key conclusions with broad implications.

Globally, litter decomposition rates were higher in home environments than in away environments. The HFA effect was significantly present in temperate, cold/highland, and subtropical climatic zones, as well as in coniferous forests, deciduous broadleaved forests, and evergreen broadleaved forests but was not detected in tropical or arid/semi-arid zones, nor in shrub or grass. The HFA effect is not determined by a single factor, but is jointly influenced by climate, litter chemical properties, and soil properties. Mean annual temperature (MAT) and soil pH showed significant negative correlations with effect size, while Litter C:P and N:P ratios were significant positive regulators. Conversely, litter carbon, nitrogen, and phosphorus contents, as well as lignin content, showed negative correlations. Among all factors, litter chemical properties (particularly total nitrogen, total carbon, and C/N ratio) explained the greatest variation in HFA effect size, surpassing the influence of climatic and soil factors. This finding supports the core theory of HFA from a plant–soil feedback perspective: plants shape their decomposer communities through litter chemical composition, and the specialization of decomposer communities in turn accelerates the decomposition of home litter, thereby forming a positive feedback loop.

Based on the above theoretical findings, our results have important practical value for forest management. In temperate, cold/highland, and subtropical regions where HFA is significant, maintaining native litter layers and promoting the growth of locally adapted tree species can accelerate nutrient cycling and enhance soil fertility. In contrast, in arid and tropical regions where HFA is not significant, active interventions, such as the use of supplemental irrigation or the introduction of complementary tree species, are likely required to offset slow decomposition rates. Furthermore, the strength of HFA varies among vegetation types (e.g., evergreen broadleaved versus coniferous forests), suggesting that species-specific litter traits and their interactions with soil microbial communities should be considered in forest restoration and plantation design. From a plant perspective, tree species selection should consider not only productivity, but also litter quality and litter–soil microbial compatibility.

In summary, this study elucidates the driving mechanisms of HFA in cross-habitat litter decomposition, providing a theoretical basis for predicting changes in decomposition rates and ecosystem carbon budgets in the context of global change.

## Data Availability

The datasets in this study are available in Supplementary Material Table S1 (data, reference).

## Supplementary Materials

The following supporting information can be downloaded at: https://www.mdpi.com/article/10.3390/plants15172716/s1. Table S1 data: Data extracted from the literature selected for inclusion in this study. Table S1 reference: List of the literature selected for inclusion in this study. The PRISMA 2020 statement (including the 27-item checklist and flow diagram) is available as supplementary material. The flow diagram is presented as Figure S1.
