# Supplementary material for: Biogeographical Differentiation and Key Drivers of the Home-Field Advantage in Cross-Habitat Litter Decomposition: A Global Meta-Analysis
Source: Plants (Basel). 2026 Sep 4;15(17):2716. doi: 10.3390/plants15172716 (PMC13567475; doi:10.3390/plants15172716)
Supplement: Supplementary file 1 [file plants-15-02716-s001.zip › Figure S1_PRISMA_flow_diagram.pdf]

PRISMA 2020 flow diagram for new systematic reviews which included searches of databases, registers and other sources

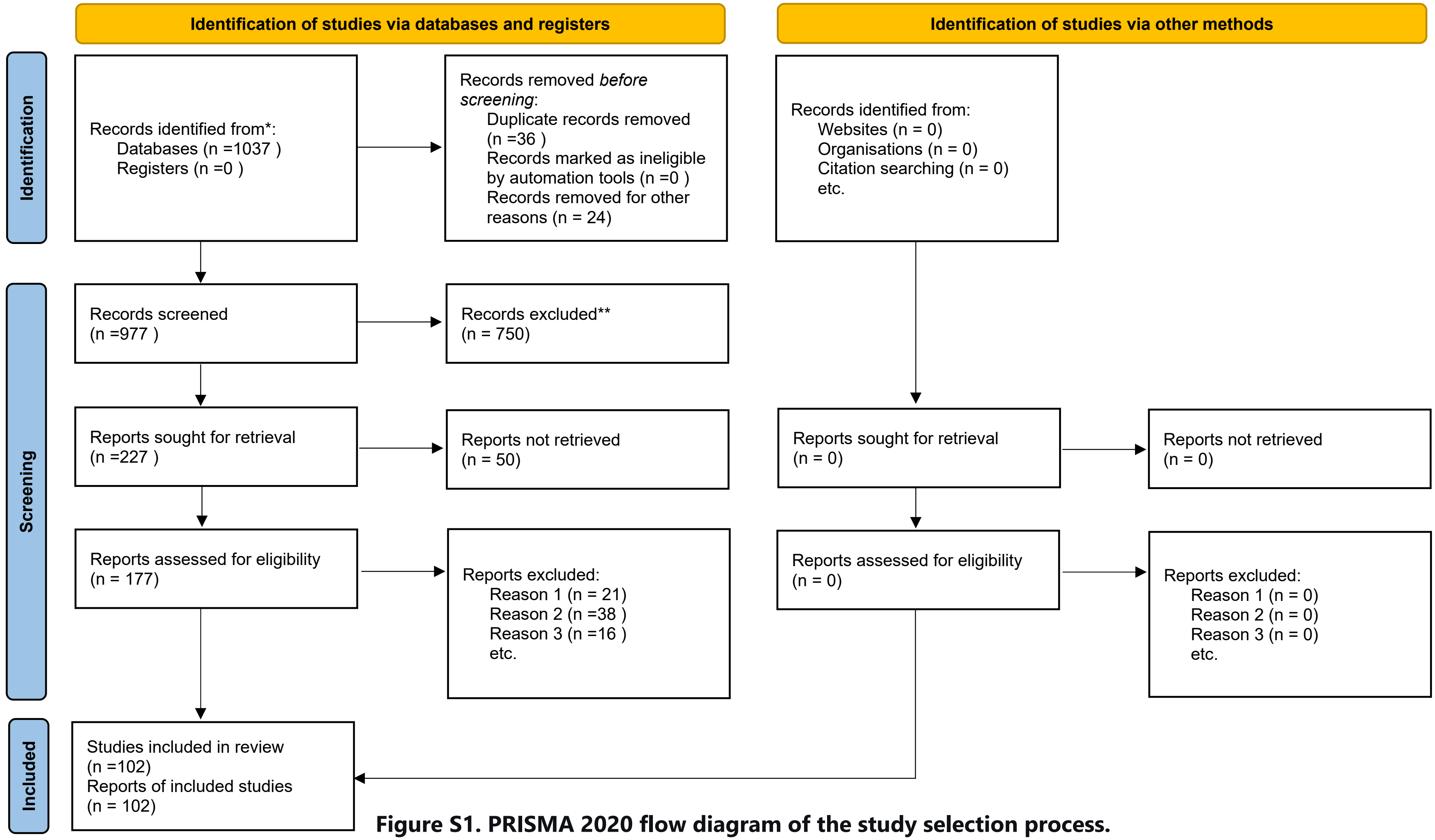

Figure S1. PRISMA 2020 flow diagram of the study selection process.

*Consider, if feasible to do so, reporting the number of records identified from each database or register searched (rather than the total number across all databases/registers).
**If automation tools were used, indicate how many records were excluded by a human and how many were excluded by automation tools.

Source: Page MJ, et al. BMJ 2021;372:n71. doi: 10.1136/bmj.n71.

This work is licensed under CC BY 4.0. To view a copy of this license, visit https://creativecommons.org/licenses/by/4.0/
